# StopSpamX: A multi modal fusion approach for spam detection in social networking

**DOI:** 10.1016/j.mex.2025.103227

**Published:** 2025-02-16

**Authors:** Dasari Siva Krishna, Gorla Srinivas

**Affiliations:** a Department of Computer Science and Engineering, Gandhi Institute of Technology and Management, Visakhapatnam, Andhra Pradesh, India; b Department of Computer Science and Engineering, Anil Neerukonda Institute of Technology and Sciences, Visakhapatnam, Andhra Pradesh, India

**Keywords:** Spammers, Hammers, Convolution neural network, Recurrent neural network, Attention-Based Models, Hybrid fusion models, Text-based classifier and Combined classifier

## Abstract

Social networking platforms like Twitter, Instagram, Youtube, Facebook, Whatsapp have completely changed people's daily routine. Users of these social media networks have total freedom to upload anything that has political, commercial, or entertainment value. The data collected from these sources can be genuine or fake. There are no concerns or problems if the data published is true and relevant. The main difficulty arises while we deal with the spam data. So, this problem of spam data should be properly handled. In order to achieve a spam free environment, researchers have proposed numerous methods and algorithms for spam detection. Out of them few algorithms are implemented to detect the spam data in twitter.

• We compare the outcomes in each scenario using various state-of-the-art word embedding techniques, such as Word2Vecv, GloVe, and FastText.
• To account for the restrictions, two deep learning hybrid fusion classifier techniques—Text-based classifier and Combined classifier—are used in this work. These classifiers are built using deep learning techniques including GRU, LSTM, and CNN.
• These methods will be evaluated using a range of measures, including F1-score, accuracy, recall, and precision. These actions could enhance the performance of the hybrid fusion approach.

## Graphical abstract

**Figure:**
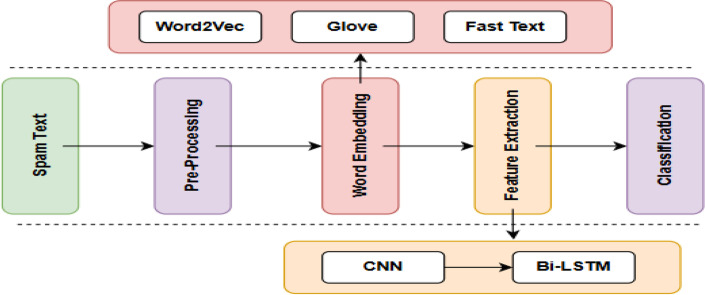

Specifications table

**Table:** 

Subject area:	Computer Science
More specific subject area:	*Machine Learning*
Name of your method:	*Text-based classifier and Combined classifier*
Name and reference of original method:	*None*
Resource availability:	*None*

## Background

In recent years, online social networks, such as Facebook and Twitter, have become an essential part of people's everyday lives. The number of social media apps is increasing day by day which resulted in a rapid growth of daily users on the internet [1]. They created a huge impact on the personal life as well as social life of a common man. Users of these social networks have total freedom to upload content that has economic, political, or entertainment value. The data generated from these social network platforms is 1.145 trillion MB per day. All the data generated on the internet is not true. There is some irrelevant or spam data [2,3] which may lead to chaos around the globe. So, this data should be properly handled. The people who post fake or spam data are called spammers. Spammers are people who are benefitted from fake data. According to latest statistics, on an average 500 million tweets are tweeted everyday which adds up to 200 billion tweets per year. As Twitter became more and more popular, spammers began to upload spam on this micro blogging service. As a result, spam detection is one of the most critical problems on the internet.

There are several deep learning techniques that can be used for text classification, includes CNNs by considering each word or n-gram as a feature in the grid, these networks may be modified to process text data. CNNs are originally developed to analyze data with a grid-like structure [4], such as an image. RNNs, including GRUs and LSTM networks [5], are designed to handle sequential data, such as time series or natural language. In text classification, an RNN can be trained to learn a mapping from a sequence of words to a target class label. Transformers are neural network architectures that were introduced for the purpose of processing sequences, such as natural language. Transformers have been widely adopted in NLP tasks, including text classification. Attention-based models leverage attention mechanisms [6] to dynamically weigh the importance of different parts of the input sequence in making predictions. Attention-based models can be applied to text classification by learning to attend to important words or phrases in a text. Hybrid models are combine multiple deep learning techniques [7], such as combining CNNs and RNNs or transformers, to leverage the strengths of each approach.

Over the past few months, social media platforms such as Twitter in particular have become popular platforms for malicious and unsolicited content or spam, and thus the focus has been on spam detection on such platforms. Ahmad [11] have shown that combining Support Vector Machines (SVM) with user interaction features is effective for spam detection and is a behavioral analysis. Like Chinnaiah [12], they use heterogeneous feature analysis to detect spam messages using varied attributes in Twitter datasets. Work by Benevenuto [13] was earlier in the sense that it studied the user activities and message patterns to detect spammers, putting the ground for additional efforts. Fazil [14] proposed a hybrid approach which combines graph based as well as the content-based techniques for blocking automated spammers more effectively. In their work, Colladon [15] further expanded discussion of spam impact by quantifying the spam's effects on email and Twitter networks. Also, Ban [16] pointed out the potential of using deep trained features for a Twitter spam detection and underlined the benefits of using of the advanced machine learning methods. Collectively, these studies indicate that incorporating behavioral, textual, and network-based features with sophisticated machine learning algorithms can improve the spam detection accuracy on the social platforms.

Hybrid models for text classification combine multiple deep learning techniques to leverage the strengths of each approach. Some examples of hybrid models for text classification include:

• CNNs and RNNs approaches are used to extract features from the text data, and an RNN is used to process the sequential information in the text. The output from the CNN and RNN can be concatenated and fed into a linear classifier to make the final prediction.
• Transformers and CNNs approaches are transformer model [8] is used to process the sequential information in the text, and a CNN is used to extract local features from the text data. The output from the transformer and CNN can be concatenated and fed into a linear classifier to make the final prediction.
• Attention-based models and RNNs approaches are an attention mechanism [9,10] is used to process the sequential information in the text using an RNN and to dynamically determine the relative relevance of various textual data segments. The attention mechanism and RNN can be combined and fed into a linear classifier to make the final prediction.

The work summarized as follows:

1. We demonstrate the importance of feeding the outputs of word embeddings to various Deep learning classifiers.
2. We built a novel deep learning framework that has been proved to be effective at detecting spam accounts on Twitter.
3. We then compare four deep learning classifiers CNN, LSTM, Bi-LSTM, GRU on different dimensions of GloVe, Word2Vec, FastText to find out which DL algorithm on which word embedding proved to be effective to detect spammers on twitter.
4. Finally, we tested various hybrid model in which CNN with Bi-LSTM performs better with embedding techniques.

### Related work

Despite the diversity of content and user behaviours, spam detection in social networks is a complex and evolving challenge. This area of research includes text, image, and network metadata. This section analyzes the various key studies related to the multi modal fusion approach adopted by StopSpamX.

Significant promise has been shown in the integration of multi modal data sources to improve spam detection accuracy. In a merger scheme [20] revealed the feasibility of the deep learning strategy for spam classification by combining visual, textual, and contextual features. As our understanding of spam evolves more recently, introduce multilingual and multi-modal processing using document image transformers and multilingual text encoders, adapting to the diversity and complexity of spam content introduced.

Recent studies show that sophisticated spam bot detection requires multi modal techniques. For example, a study on social bot detection (ACM Digital Library) used an adaptive homophily and heterophily (AHoHo) connection learning to enhance bot detection in social networks in 2023. Just like in the SpADe framework [21], a multi-stage, cost effective algorithm was proposed to identify spam accounts by incrementally integrating more complex features (IEEE Computer Society). Furthermore, bot detection on such platforms has been successfully driven by transformer based models using textual and behavioral data.

The text based methods are based on looking at content through natural language processing (NLP), machine learning techniques. For instance, Benevenuto [17] have early studies that involve machine learning classifiers, to find spammer on Twitter by reviews of user profiles and tweet content. Almaatouq [18] then continued these efforts by applying supervised learning by combining linguistic features into the spam detection problem of online social networks. They applied these approaches on the task of spam identification, showing the utility of text cues beyond existing methods, and highlighting the importance of using textural cues for spam detection as multimedia content grows pervasive. Based on visual features, Zhang [19] classified the spam images using convolutional neural networks (CNNs). We find that fusion based approaches that combine textual and image data have also proven effective, indicating the useful and complementary nature of these modalities in spam detection tasks. Spam detection has relied heavily in achieving good accuracy on analyzing user behavior and metadata. In a work by fake account detection in social networks is explored using user relationships, timestamps and message patterns. Graph-based methods have been subsequently leveraged to model user interactions in order to learn outlying behaviors as indicative of spamming activity.

## Method details

### Date set

Twitter 1KS − 10KN dataset Tweet column contains tweets posted by different users and type indicated whether the tweet belongs to spam or ham. We use those both columns for our analysis. Spam is represented as 1 and ham is represented as 0. It contains 11,968 rows. In that 5815 spam and 6153 rows with ham tweets. We split the total dataset into 80 % and 20 % for training and testing the models. In that training data 20 % records goes for validation.

### Methodology

• Data Preprocessing

Making every letter to lowercase Sending each tweet from the dataset to the function and passing each word to make all letters lowercase using the lower () method.

Removing special characters Removing special characters like punctuations (,), hashtags (#), at the rate (@) etc. We use string punctuation to remove these special characters so import string.

Removing stop words Should remove stop words which add no meaning to the data like to, we, from, have etc. For removing those stop words, we need to import the nltk library which already contains the “Stop word” set. After removing stop words, we need to carefully join the remaining words together.

Removing hyperlinks We use a regular expression to remove the hyperlinks in the tweets. These hyperlinks may cause ambiguity in distinguishing between spam and ham tweets.

• Word Embedding Techniques

Word embedding techniques are widely used in NLP to represent words or phrases as dense vectors in a high-dimensional space. These techniques, such as Word2Vec, GloVe, and FastText, capture semantic and syntactic relationships between words, allowing machines to understand and analyze textual data effectively. Word2Vec is a shallow neural network model that predicts word context, while GloVe leverages global word co-occurrence statistics. To handle words that are not in the lexicon and to identify morphological similarities, FastText uses subword information. These methods improve the precision and resilience of NLP models and find use in information retrieval, text classification, sentiment analysis, and machine translation.

• Deep Learning Models

Deep learning models such as CNNs, LSTM, Bidirectional-LSTM and GRU are widely used in various NLP tasks. In these Sections, We explored the basic deep learning algorithms that exemplify the strength in the base-line approaches. These algorithms performance compares with the proposed model with efficiently improves with key metrics.

## Convolutional neural networks

CNNs are primarily used for text classification and sentiment analysis tasks. They leverage convolutional layers to capture local dependencies and extract important features from input sequences. CNNs excel at capturing local patterns and are effective in tasks where the position of words matters less. The basic operations in CNN are convolution, activation function and polling. Finally it is connected to dense network. The basic mathematical operation shown in below :

### Convolution operation

The convolution procedure is a key building component of Convolutional Neural Networks (CNNs). It is employed to find objects, features, or patterns in an input data set, usually an image. During the convolution process, a small filter—also referred to as a kernel or receptive field—moves over the input data, multiplying each element by itself with the overlapping region, and then adding the results to get a single value. To generate a feature map, this procedure is carried out repeatedly across the whole input.

(1) z_j=[z_1j,z_2j,…,z_(n−h+1)j]

Where

(2) $$Z_{tj}=g(W_{\_}t\;.\;X_{\_}t\;:\;t+h-1+b_{\_}j$$

Here,

X_t: *t* + h-1 is the concatenation of word embeddings from position t to *t* + h-1,
W_j: jth filter Weight matrix,
b_j: Bias term
g: Activation function

### Activation function

The activation function is a part that gives the model non-linearities. No matter how deep a neural network is, it would act like a linear model without activation functions. Activation functions are used to provide non-linearities to data so that neural networks can recognize intricate patterns and connections.

(3) ReLU(x)=max(0,x)

### Pooling operation

CNNs require pooling operations, which are employed after convolutional layers to minimize the spatial dimensions of the input data. This lowers the number of parameters in the network and the computational complexity of the network. By removing less pertinent data, pooling helps preserve the most crucial qualities.

(4) $$Output\left(x,\;y\right)=\;max_{i=1}^{P}Max_{j=1}^{Q}Input\left(x*\;S+i,\;y*\;S+j\right)$$

Where:

• Output(*x,y*) : Pooling at position (x, y).
• *P* and *Q* : Pooling window.
• *S* : Stride

## Long short-term memory

One of the types of RNNs is LSTM (recurrent neural network which can model long term dependencies in sequential data). Its use is especially well suited for tasks that require understanding context and representing temporal relationships. Unlike the fully connected networks that we have seen up to this point, the LSTMs use a memory cell to store and propagate information over long sequences’ architecture comprises interconnected memory cells and a number of gates, including the input, forget and output gates. The model has a complex network structure that allows it to capture and learn patterns about sequential data with the long term dependencies. The Spatial feature of the image or text is learnt by the different gates exist in the LSTM. The different gates in mathematical representations as follows Forward Gate equ's [[5], [6], [7], [8], [9], [10]],

(5) ft=[σ(Wf·[ht−1,xt]+bf)]

(6) it=[σ(Wi·[ht−1,xt]+bi)]

(7) $$\overset{\cdot }{\tilde{C_{t}}}=\left[\text{tanh}\left(Wi\cdot \left[ht-1,xt\right]+bi\right)\right]$$

(8) $$Ct\;=ft\;.\;Ct-1+it\;.\;\overset{\cdot }{\tilde{C_{t}}}$$

(9) Ot=[σ(Wo·[ht−1,xt]+bo)]

(10) ht=Oi·tanh(ct)]

## Bidirectional- LSTM

Bi-LSTM is a more generalization of LSTM model which processes the input sequences through forward and backward directions. Bi-LSTM extends LSTM by considering the context from both directions, which makes it better for tasks such as named entity recognition, part-of speech tagging, machine translation. However the forward LSTM process the input sequence through from the beginning to end. It uses the same equations as the regular LSTM as shown (11), (12), (13), (14), (15), (16).

*Input Gate:*

(11) $$i_{t}^{fwd}=\left[\sigma \left(w_{fwd}^{i}\cdot \left[h_{t-1}^{fwd}1,xt\right]+b_{fwd}^{i}\right)\right]$$

*Forget Gate:*

(12) $$f_{t}^{fwd}=\left[\sigma \left(w_{fwd}^{f}\cdot \left[h_{t-1}^{fwd}1,xt\right]+b_{fwd}^{f}\right)\right]$$

*Candidate Cell State*:

(13) $$C_{t}^{fwd}=tanh\left(w_{fwd}^{c}\cdot \left[h_{t-1}^{fwd}1,xt\right]+b_{fwd}^{c}\right)]$$

*Update Cell State:*

(14) $$C_{t}^{fwd}=f_{t}^{fwd}\cdot c_{t-1}^{fwd}+\;i{\;}_{fwd}^{f}\cdot C_{t}^{fwd})]$$

*Output Gate:*

(15) $$O_{t}^{fwd}=\left[\sigma \left(w_{fwd}^{O}\cdot \left[h_{t-1}^{fwd}1,xt\right]+b_{fwd}^{O}\right)\right]$$

*Hidden State:*

(16) $$h_{t}^{fwd}=O_{t}^{fwd}\cdot \text{tanh}\;\left(C_{t}^{fwd}\right)$$

From the final stage to the beginning, the input sequence is processed by the reverse LSTM. With distinct weight matrices and bias terms, it employs formulas that are comparable to those of the forward LSTM shown in (17), (18), (19), (20), (21), (22).

*Input Gate:*

(17) $$i_{t}^{bwd}=\left[\sigma \left(w_{bwd}^{i}\cdot \left[h_{t+1}^{bwd}1,xt\right]+b_{bwd}^{i}\right)\right]$$

*Forget Gate:*

(18) $$f_{t}^{bwd}=\left[\sigma \left(w_{bwd}^{f}\cdot \left[h_{t+1}^{bwd}1,xt\right]+b_{bwd}^{f}\right)\right]$$

*Candidate Cell State*:

(19) $$C_{t}^{bwd}=tanh\left(w_{bwd}^{c}\cdot \left[h_{t+1}^{bwd}1,xt\right]+b_{bwd}^{c}\right)]$$

*Update Cell State:*

(20) $$C_{t}^{bwd}=f_{t}^{bwd}\cdot c_{t+1}^{bwd}+\;i{\;}_{f}^{bwd}\cdot C_{t}^{bwd})]$$

*Output Gate:*

(21) $$O_{t}^{\mathrm{bwd}}=\left[\sigma \left(w_{\mathrm{bwd}}^{O}\cdot \left[h_{t+1}^{\mathrm{bwd}}1,\mathrm{xt}\right]+b_{\mathrm{bwd}}^{O}\right)\right]$$

*Hidden State:*

(22) $$h_{t}^{bwd}=O_{t}^{bwd}\cdot \text{tanh}\;\left(C_{t}^{bwd}\right)$$

Concatenating the forward and backward hidden states at each time step in the equation below yields the Bi-LSTM's ultimate output in Eq's (23).

(23) $$h_{t}^{bi}=\left[\;h_{t}^{fwd}\cdot h_{t}^{bwd}\right]$$

## Gated recurrent unit

GRU is another variant of the RNN model that is computationally efficient compared to LSTM. It combines the memory cell and the hidden state into a single gating mechanism, resulting in a simpler architecture. GRU is effective in tasks that require capturing short-term dependencies in sequences.

Let *x_t_* be the input at time step *t, ht* be the hidden state at time step *t*, and *r_t_* be the reset gate at time step *t*. Similarly, *z_t_* represents the update gate.

The update equations for the GRU are as follows:

Update Gate:

(24) $$z_{t}=\sigma \left(Wz\cdot \left[ht-1,xt\right]+bz\right)$$

Reset Gate:

(25) $$r_{t}=\sigma \left(Wr\cdot \left[ht-1,xt\right]+br\right)$$

Candidate Hidden State:

(26) h∼t=tanh(Wh·[rt⊙ht−1,xt]+bh)

Hidden State Update:

(27) $$h_{t}=zt⊙ht-1+\left(1-zt\right)⊙h∼t$$

Where:

• *σ* is the sigmoid activation function.
• ⊙ denotes element-wise multiplication.
• [*ht*−1,*xt*] denotes concatenation of *ht*−1 and *xt*.
• *Wz, Wr*, Wh, *bz, br*, and *bh* are weight matrices and bias terms to be learned during training.

These equations describe the GRU processes input sequences over time, updating its hidden state and selectively passing information through the update and reset gates.

## Method validation

### Proposed model

#### Hybrid fusion model

**Fig. 1:**
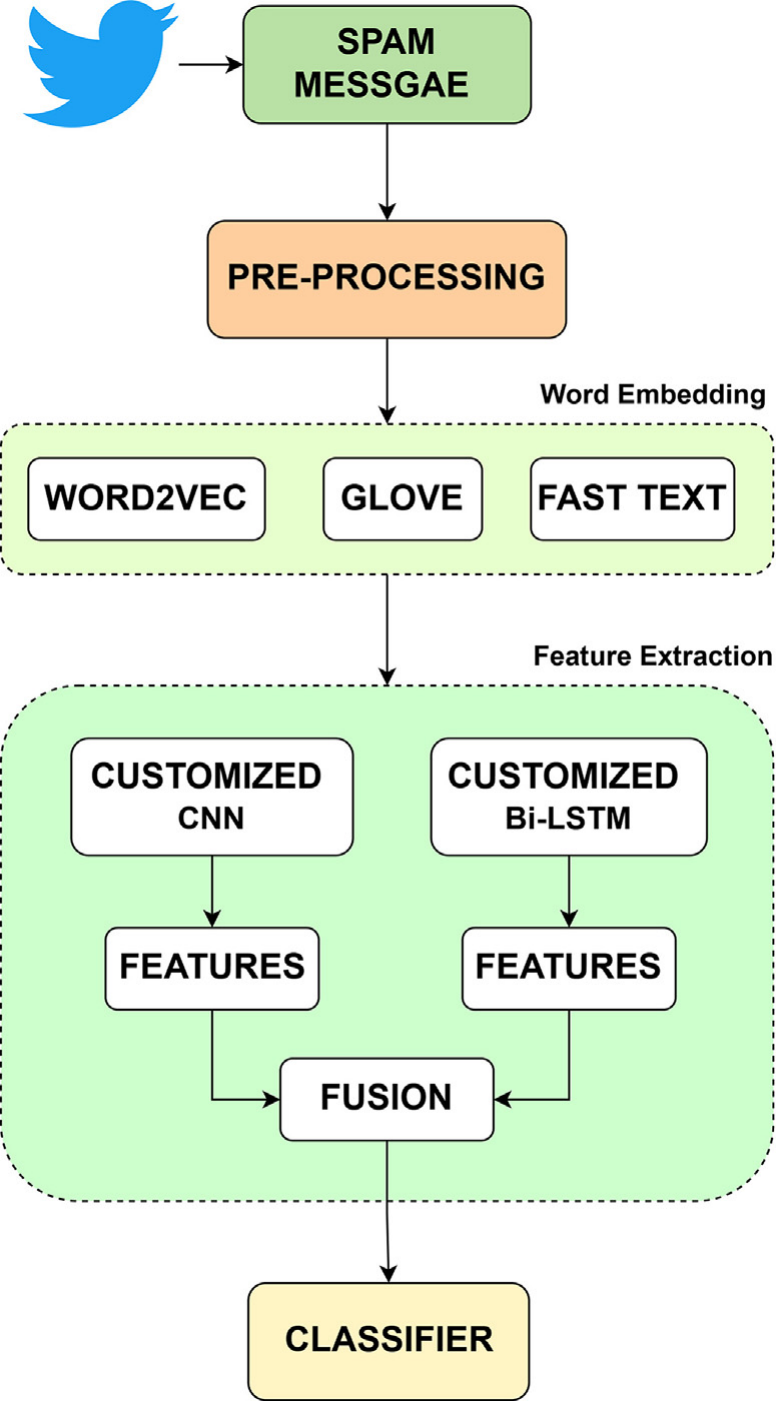
Proposed Hybrid fusion CNN + Bi-LSTM.

The proposed methodology for spam detection, as depicted in the figure [1], consists of the input comprises of spam messages collected from social networking platforms like Twitter. These messages undergo a pre-processing phase to clean and prepare the text data. Pre-processing steps typically include, Removing noise such as special characters, URLs, and unnecessary white spaces.Lowercasing all text for uniformity. Tokenizing the text into smaller units, such as words or sub-words. After pre-processing, the textual data is transformed into numerical representations using word embedding techniques. Three embedding methods are employed Word2Vec: Captures semantic meaning and context of words by mapping them to high-dimensional vectors. GloVe: Uses co-occurrence statistics to learn vector representations of words, emphasizing global context. FastText: Extends word embeddings by considering sub-word information, improving performance on out-of-vocabulary words. The embeddings ensure that the input text is converted into a format suitable for feature extraction. Two customized deep learning models are used for feature extraction. Customized CNN: Extracts local features and patterns from the embedded input. The convolutional layers capture key spatial hierarchies, such as word n-grams and local syntactic structures. Customized BiLSTM: Processes the sequential data bidirectionally, learning long-term dependencies and capturing contextual relationships from both preceding and succeeding words. Both models produce feature representations that reflect the characteristics of the input data. The features extracted by the Customized CNN and BiLSTM models are combined using a fusion mechanism. This step ensures that both local patterns (from CNN) and sequential context (from BiLSTM) are integrated, providing a richer representation of the input data Fig. 1.

By combining CNN features and Bi-LSTM features, the model can benefit from the CNN's ability to capture local patterns and the Bi-LSTM's ability to capture contextual information and long-range dependencies. This combination often leads to improved performance compared to using either architecture individually, particularly for problems where precise prediction requires both local and global knowledge.

### Experimental analysis

This section consists of evaluation measures and the comparison between the proposed approaches and existing approaches of machine learning and deep learning models.

## Evaluation measures

Standard measures, which include accuracy, precision, recall, and F1-score, are utilized for classification tasks to assess the performance of the suggested models.

i. Accuracy(Acc): It is calculated by dividing the total number of predicted messages by the number of messages that were accurately anticipated.

Acc= $\frac{(TP\;+\;TN)\;}{\;(TP\;+\;FP\;+\;FN\;+\;TN)\;}$

ii. *Precision(Pre):It is the percentage of proportion of positive predictions (Spam) that are truly positives.*

Pre= $\frac{TP\;}{\;(TP\;+\;FP)}$

iii. *Recall (Recall): It is the percentage of proportion of actual Positives that are correctly classified.*

Recall = $\frac{TP\;}{\;(TP\;+\;FN)\;}$

iv. *F1-Score(F1): It is the harmonic mean of precision and recall.*

F1 − Score = $\frac{2\;\times \;Precision\;\times \;Recall\;}{\;Precision+Recall\;}$

## Experimental results

In this study, we applied various deep-learning methods to different word embeddings to classify tweets as Spam or Not-Spam. Word2Vec, GloVe, and FastText were used as word embedding techniques. We maintained the same data distribution across all algorithms for training and testing: 80% for training and 20% for testing. For Word2Vec, we used a pre-trained corpus. Similarly, GloVe was based on pre-trained English word vectors trained on the 2014 Wikipedia + Gigaword 5th Edition corpora (6B tokens, 400K vocabulary). Likewise, we also used a pre-trained corpus for FastText.The dataset was preprocessed and embedded with word vectors of different dimensions: 50-dimensional, 100-dimensional, and 200-dimensional pre-trained word vectors. The experimental results of different deep learning models with these embedding techniques are shown in Table 1, Table 2, Table 3, respectively.

Discussion: In this investigation, the dataset was trained with word2vec word embedding with different pre trained word dimensional vectors such as 50, 100 and 200. Out of all tested models LSTM preformed better than the other deep learning techniques. The proposed model out performed compared with the other deep learning approaches. The proposed model with word2vec word embedding shown 97.20, 97.85, 98.48 with 50,100,200 dimensions respectively as shown in Table 1.

**Table 1:** WORD2VEC word embedding.

Model	Word2vec with 50 dimensions				Word2vec with 100 dimensions				Word2vec with 200 dimensions			
	Acc	Pre	Recall	F1	Acc	Pre	Recall	F1	Acc	Pre	Recall	F1
GRU	93.90	93.97	93.94	93.95	93.48	93.71	93.43	93.55	91.64	91.75	91.74	91.74
LSTM	94.73	94.87	94.68	94.74	93.60	93.67	93.66	93.62	94.23	94.34	94.19	94.24
Bi-LSTM	94.11	94.15	94.13	94.14	93.27	93.37	93.38	93.31	93.19	93.30	93.22	93.24
CNN	92.27	92.44	92.18	92.24	93.31	93.53	93.22	93.29	92.39	92.52	92.32	92.37
Proposed Work	97.20	97.80	97.50	97.40	97.85	97.90	97.80	97.90	98.48	98.80	98.20	98.40

Discussion: In this investigation, the dataset was trained with Glove word embedding with different pre trained word dimensional vectors such as 50, 100 and 200. Out of all tested models GRU preformed better than the other deep learning techniques. The proposed model out performed compared with the other deep learning approaches. The proposed model with word2vec word embedding shown 96.68, 96.54, 96.18 with 50,100,200 dimensions respectively as shown in Table 2.

**Table 2:** Glove word embedding.

Model	Glove with 50 dimensions				Glove with 100 dimensions				Glove with 200 dimensions			
	Acc	Pre	Recall	F1	Acc	Pre	Recall	F1	Acc	Pre	Recall	F1
GRU	95.07	95.19	95.02	95.06	94.92	94.52	94.54	94.52	94.65	94.77	94.60	94.64
LSTM	94.19	94.20	94.18	94.19	94.82	94.94	94.77	94.81	94.52	94.52	94.53	94.52
Bi-LSTM	94.44	94.43	94.45	94.44	93.94	93.94	93.93	93.94	93.85	93.85	93.86	93.85
CNN	93.69	93.80	93.52	93.70	93.60	93.75	93.58	93.59	93.85	93.91	93.84	93.79
Proposed Work	96.68	97.42	96.90	96.54	96.54	96.13	96.59	96.10	96.18	96.19	97.21	96.80

Discussion: In this investigation, the dataset was trained with fast text word embedding with different pre trained word dimensional vectors such as 50, 100 and 200. Out of all tested models GRU preformed better than the other deep learning techniques. The proposed model out performed compared with the other deep learning approaches. The proposed model with word2vec word embedding shown 94.40, 94.80, 95.20 with 50,100,200 dimensions respectively as shown in Table 3.

**Table 3:** Faste text word embedding.

Model	Fasttext with 50 dimensions				Fasttext with 100 dimensions				Fast text with 200 dimensions			
	Acc	Pre	Recall	F1	Acc	Pre	Recall	F1	Acc	Pre	Recall	F1
GRU	92.05	92.09	92.08	92.07	93.60	93.67	93.66	93.62	93.94	93.85	93.85	93.86
LSTM	91.01	91.78	91.75	91.09	91.64	91.75	91.74	91.74	92.27	92.44	92.18	92.24
Bi-LSTM	91.58	91.58	91.65	91.55	92.27	92.44	92.18	92.24	94.44	94.43	94.45	94.44
CNN	90.98	91.02	90.99	90.95	92.27	92.44	92.18	92.24	93.85	93.85	93.86	93.85
Proposed Work	94.40	94.28	92.90	93.49	94.80	94.40	94.60	94.80	95.20	94.90	94.50	94.80

## Comparison study

**Fig. 2:**
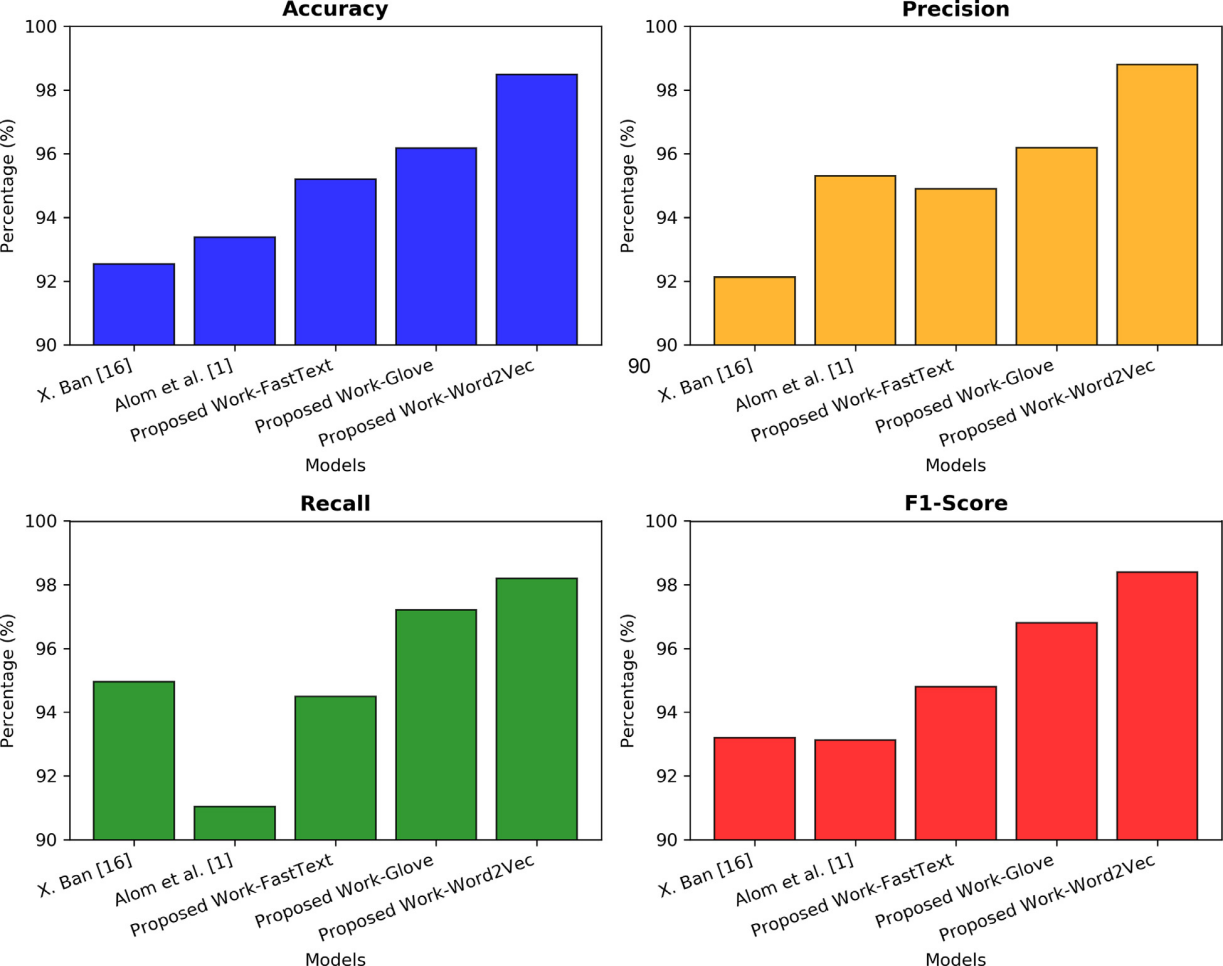
Comparison of performance matrices.

In this investigation, we compared the exiting results with other state of art models. In the proposed model we investigated the deep fusion mechanism with different word embedding dimensions such as 50, 100 and 200. These Dimensions in contextualization of words plays a critical role with various embedding techniques such as word2vec, glove and fasttext. The Table 4 shows the improved results of various existing DL algorithms with our proposed model and the detailed comparison shown in Fig. 2.

**Table 4:** Comparison of proposed model with existing deep learning models.

Models	Acc	Pre	Recall	F1
X. Ban [16]	92.53	92.13	94.96	93.2
Alom et al. [1]	93.38	95.31	91.04	93.12
Proposed Work- FastText	95.20	94.90	94.50	94.80
Proposed Work- Glove	96.18	96.19	97.21	96.80
Proposed Work- Word2Vec	98.48	98.80	98.20	98.40

## Conclusion

In order to solve the present shortcomings of deep based spam detection approaches, we have introduced deep learning methods based on the Twitter spam detection methodology in the present investigation. We developed a text-based classifier that only assesses tweets written by people. We demonstrated in the studies that combined CNN with Bi-LSTM outperforms comparing with other deep learning-based alternatives. The hybrid model outperforms existing strategies for categorizing tweets into spam or ham, according to the experimental evaluation of the suggested strategy. The Twitter platform can be transformed into a spam free environment by reducing the dangers associated with smashing attacks on the internet. This technology can dramatically improve the security of all the 217 million twitter users across the world. This research addressed the difficulty of identifying social network spam and the requirement for choosing the optimal word embedding to accurately reflect the spammer behavior without information loss owing to the increase in incoming data caused by online spammers.

## Limitations

Not Applicable.

## CRediT author statement

Dasari Siva Krishna conceived the idea for the study and was responsible for the design and development of the multi-modal fusion framework for spam detection. They led the implementation of the spam detection algorithm, conducted the experimental analysis, and wrote the majority of the manuscript.

Gorla Srinivas contributed to the data collection and preprocessing, as well as the fine-tuning of deep learning models. They supported the analysis and interpretation of results and provided critical feedback on the manuscript, including the validation of the proposed approach.

## Declaration of competing interest

The authors declare that they have no known competing financial interests or personal relationships that could have appeared to influence the work reported in this paper.

## Data Availability

Data will be made available on request.
